# Clinical Characteristics and Prognosis of Primary Tracheal Cancer: A Single Institution Experience

**Published:** 2018-10-01

**Authors:** Rashmi Koul, Reem Alomrann, Shrinivas Rathod, Julian Kim, Ahmet Leylek, Naseer Ahmed, Bashir Bashir, Amitava Chowdhury, Lawrence Tan, Arbind Dubey

**Affiliations:** 1 Department of Radiation Oncology, Cancer Care Manitoba, University of Manitoba, Winnipeg, Manitoba, Canada; 2 Section of Thoracic Surgery, Department of Surgery, Faculty of Health Sciences, University of Manitoba, Winnipeg, Manitoba, Canada

**Keywords:** Primary tracheal carcinoma, Tracheal tumours

## Abstract

**Background **Primary tracheal cancers (PTCs) are rare and current evidence-based understanding is limited to retrospective reports and national databases. We present single institutional study of a historical cohort of PTC from Canadian provincial cancer registry database.

**Materials and Methods: **After institutional research ethics board approval, all PTC patients diagnosed from 1980 to 2014 were identified through the Canadian provincial cancer registry. Demographic and tumor related factors were evaluated using descriptive statistics. Survival rates were estimated using the Kaplan-Meier method and cox hazard regression analyses were performed to identify predictors of disease-free survival (DFS) and overall survival (OS).

**Results: **A total of 30 patients were included in the study. At presentation, 10 patients (33%) had only local disease, 14 patients (47%) had locoregional disease and the remaining 4 patients (13%) had distant metastasis. The majority of patients underwent primary radiation treatment. The overall survival rate was 30% at 2 years and 16% at 5 years. Patients receiving radical-intent therapy had better 2-year DFS and OS compared to patients managed with palliative radiotherapy and best supportive care (46%, 17% and 0%) (p=<0.001) and (50%, 23% and 0%) (p=<0.001), respectively. Radiotherapy resulted in a better 2-year OS and DFS (32% versus 14%) (p=<0.03) and (32% versus 0%) (p=<0.001), respectively.

**Conclusion: **PTC is an uncommon neoplasm making the study of the disease technically and logistically challenging. Radical radiotherapy alone is curative option in inoperable PTC. Intent of treatment and radiotherapy were associated with superior survival outcomes.

## Introduction

 Tracheal tumors are rare clinical entities, accounting for 0.1 to 0.4% of all malignant tumors found in contemporary populations^   1 ^. Tracheal tumors arise most often from the respiratory epithelium of the trachea, salivary glands and mesenchymal structures. The majority of tracheal tumors are malignant amongst adults (80–90%); however, pediatric presentations are often benign (60–70%) ^2^^,^^3^ . Literature regarding pathologic distribution of the primary tracheal cancers (PTCs) is sparse and little information is available about their natural history and behavior in literature^   4 ^.

Due to the rarity of this disease, randomized studies are not feasible and current evidence-based understanding is limited to retrospective reports and national databases. This is a retrospective review of a provincial cancer registry database to describe patients, treatments, and outcomes of PTC in Manitoba, Canada.

## MATERIALS AND METHODS

 Cancer Care Manitoba is the tertiary cancer center in the Canadian province of Manitoba, serving a catchment population of approximately 1.3 million people. After institutional research ethics board approval, all PTC patients diagnosed from 1980 to 2014 were identified through the provincial cancer registry. During the study period, the care of PTC patients was done in the setting of a multidisciplinary oncology team who managed these patients according to institutional practice guidelines. 

Patient data, including demographics, clinical and pathological characteristics (presenting symptoms, smoking history, histological diagnosis, location, extent of disease) and treatments (intent, modality) were abstracted from review of both paper and electronic medical records. The extent of the disease was defined as follows: Tumors were defined as local if the tumor was confined to the trachea, locoregional if the tumor extended beyond the trachea to the adjacent structures and or metastasized to the mediastinal or supraclavicular lymph nodes, and metastatic if the tumor spread to distant organs.

Disease-free survival (DFS) was defined as the time interval from diagnosis to recurrence, death, or loss to follow-up. Overall survival (OS) was calculated from the date of diagnosis to the date of last follow-up or death. Demographic and tumor- related factors were evaluated using descriptive statistics. Survival rates were estimated using the Kaplan-Meier method and were compared using a two-sided log-rank test. Univariate hazard regression analyses were performed in order to identify predictors of DFS and OS using Cox proportional hazards models. A p-value of 0.05 was considered statistically significant.

## Results

**Patient characteristics**

 From 1980 to 2014, a total of 30 newly diagnosed PTC were identified, of whom 20 were males and 10 were females (Table 1). The mean age at the time of diagnosis was 68 years (range 39-90). Eighty percent of the patients (n=24) were either active or remote smokers with a minimum of 13 pack- year history of smoking. Cough (67%) and dyspnea (63%) were the most common presenting symptoms reported by patients. 

At presentation, 10 patients (33%) had local disease, 14 (47%) had loco-regional disease with extension to an adjacent organ and or mediastinal and or supraclavicular lymphadenopathy, and 4 patients (13%) had distant metastasis. Tumor location varied among patients with 8 in upper 1/3, 6 in middle 1/3, and 13 involving with distal tumor. Location details were not available for 3 patients. 

Squamous cell carcinoma (47%), small cell carcinoma (20%) and adenoid cystic carcinoma (10%) were the most common pathological subtypes of PTC. The baseline demographics and clinical characteristics of the patients are tabulated in Table 1. 

**Table 1:** Demographic and tumor details

**Characteristic**	**No. of patients (%)**
Gender (Male:Female)	2:1
Mean age	68 years (39-90)
Presenting symptoms	
Cough	67%
Dyspnea	63%
Hemoptysis	43%
Stridor	30%
Hoarseness	20%
Histopathology	
Squamous cell carcinoma	14 (47%)
Small cell cancer	6 (20%)
Adenoid cystic carcinoma	3 (10%)
Others	5 (17%)
Smoking History	
Yes	24 (80%)
No	1 (3%)
Extent of disease	
Local only	10 (33%)
Locoregional	14 (47%)
Metastatic	4 (13%)

**Treatment details**

Amongst the 30 patients, 2 underwent primary surgical resection followed by adjuvant radiotherapy to receive 60 Gy in 30 fractions, 10 received definitive radiotherapy (>50Gy) and 12 received palliative radiotherapy. Three patients received palliative chemotherapy. Five patients were treated with best supportive care (BSC).

**Survival Outcomes and prognostic factors**

**Figure 1:**
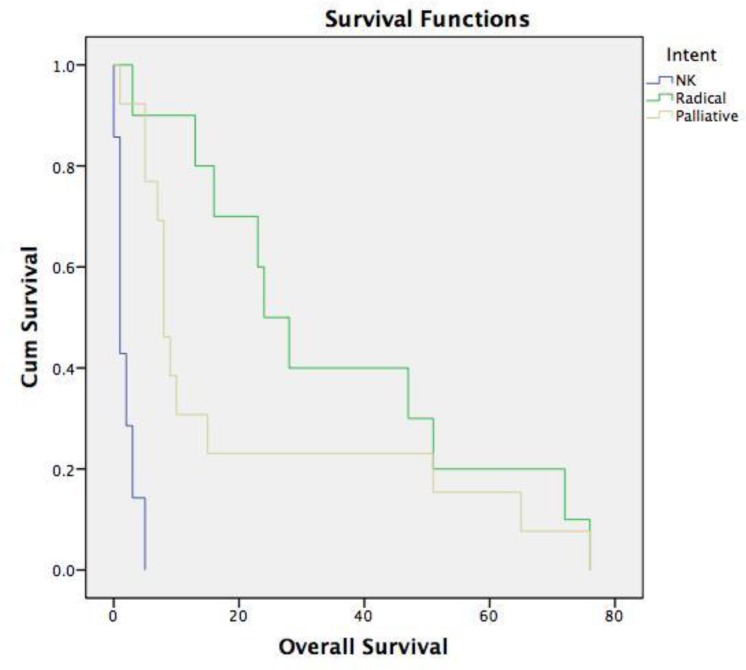
Kaplan-Meier plot of estimated 2-year survival in patients who received radical intent therapy (green line), palliative intent therapy (yellow line) and best supportive care (blue line).

The median survival for the entire cohort was 9 months with a two-year survival estimate of 30%. Univariate hazard regression revealed treatment intent and radiotherapy were both statistically significant predictors for both disease-free survival and overall survival (Table 2). Patients who received radical-intent therapy, including surgery and radiotherapy had better 2-year DFS and OS compared to patients managed with palliative radiotherapy and best supportive care (46%, 17% and 0%) (p=<0.001) and (50%, 23% and 0%) (p=<0.001), respectively (Figure 1). Radiotherapy resulted in a better 2- year OS and DFS (32% versus 14%) (p=<0.03) and (32% versus 0%) (p=<0.001), respectively. Extent of disease was not associated with DFS or OS (p=0.14). Predictors of DFS and OS are presented in Table 2.

**Table 2:** Prognostic factors

**Prognostic factors**	**2- year DFS**	**P- value**	**2- year OS**	**P- value**
Extent of disease				
Local	27%	0.24	38%	0.14
Loco regional	29%		27%	
Metastatic	0%		0%	
Intent				
Radical	46%	<0.001	50%	<0.001
Palliative	17%		23%	
Best supportive care	0%		0%	
Radiotherapy				
Yes	32%	<0.001	32%	<0.03
No	0%		14%	

## Discussion

 In this study, we present outcomes of PTC treatment and prognostic factors obtained from a Canadian provincial cancer program. Primary carcinoma of the trachea is an uncommon neoplasm, making the study of this disease technically and logistically challenging. Furthermore, data related to these tumors are mostly confined to retrospective studies or case series. 

There is a significant heterogeneity in pathological distribution and outcomes noted in the reported literature. Squamous-cell carcinoma (SCC) and adenoid cystic carcinomas (ACC) have been reported to comprise about two-thirds of adult primary tracheal tumors ^2^^,^^5^ . ACC patients have a younger age at presentation (5^th^ decade of life over SCC that present typically in 6^th^ decade of life). The rest third of PT tumors consisted of a widely heterogeneous group of malignant tumors. In our cohort, the mean age was 68 years. The most common symptoms reported in the literature are hemoptysis, dyspnea, cough, hoarseness and stridor^   6 ^. In our series, cough was the predominant symptom in keeping with the literature. Smoking was common amongst our cohort in keeping with the other published literature^   2 ^. Computed tomography (CT) is the commonly used and the radiological appearance of the tumors can be classified as intra-luminal, wall thickening and exophytic^   7 ^. Endoscopic evaluation reveals that the majority of the lesions are bulky and obstructive in nature ^8^^,^^9^ . 

There is no standard AJCC staging system for PTC. Although individual groups have proposed staging systems, these systems are not widely used^   8 ^. In this study, patients were grouped as local, loco-regional or metastatic depending on the nodal and metastatic extent of disease. In our study, 10 (33%) patients had local disease, 14 (47%) had loco- regional disease and 4 (13%) had metastatic disease. 

Due to the rarity of PTC, there are no prospective randomized clinical trials or large prospective series. Thus, the choice of management and the optimal sequence of treatment remain undefined. Surgery is the preferred treatment^        10 ^, and with modern surgical techniques resectability rates have improved and almost half in PTC could be resected ^11^^-^^14^ . Complete resection is the desired goal and demands knowledge of the principles of tracheal surgery^   15 ^. Interventional endoscopy and endo-luminal brachytherapy could also serve as a potential alternative^   16 ^. Radical radiotherapy in inoperable cases may represent a potentially curative treatment option^   17 ^. 

Lymph node involvement or invasive margin positivity is associated with an adverse effect on the management of SCC; such an effect is not demonstrable with adenoid cystic carcinoma. Adjuvant radiotherapy could further improve disease-specific and overall survival rates^   18 ^. After surgical management, the 3- and 5-year survival estimates are 80%, 48% for ACC, 80% and 20% for SCC^   19 ^. With surgical resection, 5-year survival rates of 40-60% are reported, but with radiation lower survival rates (5-year OS 6-11%) are noted. This could be possibly related to case selection bias and the higher percentage of ACC in surgical series. 

Radical radiation is a viable option for incompletely resected or unresectable PTC in both radical and palliative settings. In our series, treatment median survival for the entire cohort was 9 months with a 2-year survival estimate of 30%. Our study found that treatment intent was a significant predictor of improved OS and DFS, albeit at the univariate level. Patients treated with radical intent treatment had significantly better 2-year OS (50% vs 23% vs 0%; p<0.001) compared with those treated with palliative treatment or best supportive care, respectively. Similarly, the 2-year DFS (46% vs 17% vs 0%; p<0.001) was greater in patients treated with radical-intent treatment compared to those treated with palliative treatment and best supportive care, respectively. Univariate analysis showed the use of radiation was a significant predictor for improved OS (2- year rate 32% vs 14 %; p<0.001) and DFS (2- year rate 32% vs 0%; p<0.001). No significant difference was observed in the majority of patients with PTC undergoing primary radiation-based treatment. 

There are several limitations to our study. Since the study period spans 34 years, from 1980 to 2014, our study is subject to stage migration biases as well as differences in surgical and radiotherapy techniques that could not be accounted for the limited size of the cohort. Due to the retrospective nature of this study, missing disease and treatment-related details could have resulted in misclassification of patient characteristics or outcomes. Data collection and reporting could offer additional information and may help to optimize the treatment approach for PTC. 

## CONCLUSION

 Primary tracheal carcinoma is an uncommon neoplasm that makes the study of the disease technically and logistically challenging. In PTC, radiotherapy alone is a possible curative treatment option in inoperable cases. Intent of treatment and radiotherapy were associated with superior survival outcomes. 
